# An introduction to the Item Response Warehouse (IRW): A resource for enhancing data usage in psychometrics

**DOI:** 10.3758/s13428-025-02796-y

**Published:** 2025-09-05

**Authors:** Benjamin W. Domingue, Mika Braginsky, Lucy Caffrey-Maffei, Joshua B. Gilbert, Klint Kanopka, Radhika Kapoor, Hansol Lee, Yiqing Liu, Savira Nadela, Guanzhong Pan, Lijin Zhang, Susu Zhang, Michael C. Frank

**Affiliations:** 1 Stanford University, Stanford, CA USA; 2 Harvard University, Cambridge, Massachusetts USA; 3 New York University, New York, USA; 4 The University of Edinburgh, Edinburgh, UK; 5 University of Illinois Urbana-Champaign, Urbana-Champaign, Illinois USA

**Keywords:** Psychometrics, Big data, Item response theory, Measurement, Factor analysis

## Abstract

The Item Response Warehouse (IRW) is a collection and standardization of a large volume of item response datasets in a free and open-source platform for researchers. We describe key elements of the data standardization process and provide a brief description of the over 900 datasets in the current iteration of the IRW (version 28.2). We describe how to access the data through both the website and an API, and offer a brief tutorial with example R code illustrating how to download data from the IRW and use it in standard psychometric analyses. While we are continuing to develop the IRW, this presentation may help researchers utilize data from this resource for work in psychometrics and related fields.

## Introduction

The Item Response Warehouse (IRW) is a large collection of publicly available psychometric datasets standardized to a common format for the purposes of providing data for future research on psychometric methods. This effort is aligned with the growth of standardized data resources in other disciplines that have supercharged subsequent research. For example, in computer science, the introduction of ImageNet (Deng et al., 2009), a large-scale dataset of images, revolutionized AI by providing a critical benchmark for training and evaluation.

While the IRW uses previously available data, the articulation of a data standard and harmonization of data resources to meet that standard is a useful development for both methodologists and applied researchers in psychometrics, mathematical psychology, and related disciplines in the social and behavioral sciences. Similar data standards have been adopted in other fields such as genetics and neuroscience (Purcell et al., 2007; Gorgolewski et al., 2016), and we propose that the IRW would be equally useful in psychometric research. Further, making these data resources available in a single location with accompanying software increases the degree to which they are findable, accessible, interoperable, and reusable (i.e., FAIR; Wilkinson et al., 2016).

The data resources of the IRW are available via both a website and programmatic access via API. We describe the types of data we include and the structure we use for standardization. The IRW already contains over 2 billion item responses in a standardized format. While some of this data is idiosyncratic, large tranches of it could be easily analyzed in widely used psychometric software such as mirt, lme4, lavaan, brms, psychonetrics, and others (Chalmers, 2012; Rosseel, 2012; Bates et al., 2014; Bürkner, 2021; Epskamp and Rcpp, 2020; Isvoranu et al., 2022); we illustrate how this can be done with brief tutorials. We also describe a number of metadata elements that we have developed, allowing users to filter through the various IRW datasets to identify those with characteristics relevant to them. While we aim to continue to grow the IRW and to increase interoperability with other computational tools, it is already at a stage where it could help improve the rigor and impact of current research.

## The IRW

The IRW consists of both a collection of ‘tables’ and a system of technological infrastructure that facilitates their acquisition.^1^ We first describe the data resources before a description of how users can access the IRW. Our discussion of the IRW begins with a consideration of what data we focus on for inclusion in the IRW.

### Inclusion criteria

There is a wide range of data available in the IRW derived from a wide variety of sources. For example, we have included numerous data sets from popular R packages focused on psychometrics (e.g., Mair, 2018; Robitzsch and Steinfeld, 2018; Van der Ark, 2012; George et al., 2016; Robitzsch et al., 2022b; Heller and Wickelmaier, 2013; Robitzsch, 2022a). We also include data from papers that have made data publicly available, educational technology firms that have posted data, etc. Our aim is to collect as much relevant data as possible, and to that end, we have a list of data resources that we plan to target in future collection efforts.

One critical issue that we have aimed to manage in building the IRW pertains to data licensing. In particular, the IRW complies with the licensing requirements under which the data were originally made available. Alongside information about licenses, we also describe the provenance of data (along with tools on the website meant to ease the process of citing the original data) and provide documentation linking the IRW-compliant version of the dataset that we have produced. Finally, the code used to produce IRW-compliant versions of the dataset is publicly available.^2^

Inclusion criteria for IRW are quite broad and nonspecific. This is by design, given that there are common approaches available for analyzing and understanding a broad range of psychometric data types (Takane and De Leeuw, 1987; Zhang and Chen, 2022). Making more expansive sets of data available to researchers will be beneficial so long as the IRW is structured so that researchers can easily select subsets of data that are relevant for their purposes. For inclusion in the IRW, the core requirement is that the data contain cross-classified responses. By responses, we emphasize that the IRW contains the constituent elements of a psychological measure – the item responses – rather than just the aggregate. For example, data containing only the scale scores of students on some standardized test would not be admissible; the data would need to contain the item responses from which those scale scores were computed. Further, note that the responses are required to be (1) real (the IRW does not contain simulated data)^3^ and (2) pertaining to a latent variable or other psychological construct. We thus exclude datasets that solely consist of measures of manifest variables, such as physical attributes (e.g., length) or biomarkers (e.g., heart rate).

The second key requirement is that responses be cross-classified. Responses need to be classified by both a (1) measurement object (e.g., individuals) and a (2) measurement instrument (e.g., items; note that we require that there be more than one item used, so single-item scales are not included). This basic template captures a broad variety of data related to educational testing, survey data including personality and health surveys and Likert-style responses (Likert, 1932), and many other settings. Data that meet these requirements are the key inputs for a wide range of latent variable models in psychometrics and related fields, including item response theory (van der Linden, 2016), Bayesian knowledge tracing (Pelánek, 2017), generalizability theory (Shavelson et al., 1989), factor analysis (Gorsuch, 2014), structural equation modeling (Bowen and Guo, 2011; Kline, 2023), network psychometrics (Epskamp et al., 2017; Isvoranu et al., 2022), and multilevel or cross-classified modeling (Rabe-Hesketh and Skrondal, 2008), among others.

While the canonical case in psychometrics is that of persons responding to items, there are other scenarios that we include in the IRW. For example, legislators’ voting records on individual bills are of interest for creating indices of political partisanship and would meet our data requirements. Similarly, multiple judges scoring the performance of an athlete in a figure skating or gymnastics competition are eligible (given that responses for a single athlete are cross-classified across raters). In many instances, a person is the focus of measurement, but this need not always be the case. For example, persons (acting as the instruments) rating faces (the object of measurement) are included (Chen et al., 2021). We emphasize the importance of persistent identifiers. For example, movie reviews (in the form of a number of stars) from multiple individuals (where the movie is the object being measured and the persons are the instrument) would not meet our criteria unless the reviews contain a persistent identifier of the rater.

The IRW’s breadth in including such a variety of data is by design. This breadth means that the IRW needs to allow users to carefully select tables meeting certain specifications (e.g., users may wish to exclude data that contains repeated trials of an item). However, we argue that this richness of data will ultimately prove beneficial. Larger volumes of data mean that an end user has more options if they want to study some technique in a wide variety of datasets. Further, there are good reasons to suspect that the introduction of non-standard data may lead to methodological innovations via ‘borrowing’. For example, as educational measurement becomes more focused on the problems associated with the measurement of ability in longitudinal systems, we may glean insights using techniques from related fields. As an illustration of how such borrowing may work, techniques used to understand the evolving ideologies of judges (Martin and Quinn, 2002) may offer a methodological template for analysis outside of political science.

### The IRW data standard

Once data have been deemed eligible for inclusion in the IRW, they are processed to ensure compliance with the IRW data standard. This data standard is described in detail at the IRW website; we emphasize the key elements here.^4^ After describing these key elements, we turn to a variety of ancillary data that we have also included in a standardized format when available.

We note one important fact about our processing of IRW data. We have generally worked to create tables for the IRW that focus on single constructs. For example, if a single study separately measures anxiety and depression, this will result in two tables in the IRW. Some researchers may wish to further split the tables in the IRW, and some may wish to combine tables.^5^

#### Additional data elements

While the IRW contains a wide variety of data, all tables contain a few core data elements as a function of our inclusion criteria. The core data elements of the IRW are threefold; we offer a small illustration in Table 1. First, an identifier focusing on the unit that the instrument is attempting to measure (the object of measurement, frequently a person). Second, an identifier of the measurement instrument (frequently an item). Third, a response that is scored so that it can be treated as ordinal. In many cases, this is a matter of coding correct or affirmative responses as 1 and incorrect or disaffirming responses as 0. Many other datasets involve scores, such as the number of points a student might get for a partial credit item. In some cases, the response may be continuous (e.g., the user-selected position of a slider bar). No matter the nature of the response, we have taken care to ensure that it is coded so that the values are meaningful (e.g., numeric values can be treated as ordinal). Note also that, as collected in IRW, the data are in ‘long’ form and each row contains information about a single item response.^6^ The elements of Table 1 are sufficient for many forms of analysis, but in other cases, additional data will be available. We turn now to a discussion of these ancillary data elements.

**Table 1:** Basic long structure of IRW data

id	item	resp
1	1	0
2	1	1
1	2	0
2	2	1

#### Other key ingredients

Alongside the elements of Table 1, we have included other select data when available. In general, these data elements are specific to the dataset and additional information can be obtained from the references in the IRW dictionary. However, some data elements are sufficiently common to merit standardized formatting. We comment on those here (with their name in the standardized tables in parentheses):

- Response time (rt): The reported time to produce an item response, when available, is coded in seconds.
- Date (date): The calendar time at which a response was produced is included. Coding of this variable is done in two ways. In some cases, there were only relative dates (e.g., 30 days into data collection). In that case, we convert to the number of seconds since the first piece of data in the dataset was collected). In cases where more exact information was given (e.g., 1:30 PM 03/04/2008), we convert to Unix time (i.e., seconds since JAN 01 1970. (UTC)).^7^ In both cases, the date is available for analysis as a straightforward numeric quantity; in particular, differences and order would be easily ascertainable.
- Rater (rater): In scenarios wherein the focal unit is being rated by other observers, an identifier associated with the rater that produced the response is recorded so that rater effects can be studied.
- Q Matrix loadings (qmatrix__*): When items are classified into a small number of skills (i.e., for the purposes of cognitive diagnostic modeling; Ravand and Robitzsch , 2015), we have included these item-level classifications.
- Treatment indicators (treat): When data come from a study with some causal focus, we indicate responses from treated subjects with values of 1 (and control as 0).
- Wave (wave): Wave is recorded in the cases of longitudinal data collection that is punctuated and not continuous (in which case, date would be more appropriate).
- Covariates (cov_*): Covariates associated with id, such as age and gender, are flagged so that they are available for study of, for example, differential item functioning (Camilli, 2006).

In some cases, the IRW contains relatively few tables that contain these additional elements, but we aim to target these areas for future growth. Note that the data standard is evolving as we are identifying new features of data that can be standardized in the IRW.

#### IRW metadata

We track a variety of metadata elements to help researchers identify and select tables of interest. We describe these separately as quantitative (which are metadata elements that are calculated based on the tables themselves) and qualitative (which are based on human annotation) metadata elements. The quantitative metadata are straightforward; for example, the number of responses, the number of measurement probes, and the number of objects that are measured. We also compute density (as defined below) for each table, as different values of density often require specialized handling. The qualitative metadata describe the measurement tool (e.g., is the tool measuring a cognitive construct? an affective construct?) and the sample (e.g., when the objects being measured are humans, what is the age range of these respondents?). The metadata are critical in that they allow IRW users to rapidly filter the large set of IRW tables down to the specific ones that meet their requirements (e.g., using irw::irw_filter(), see Section “Programmatic work with IRW data in R”).

### Using the IRW

The IRW data resources are available via several mechanisms. We first describe the IRW website, which is a key component that documents the data standard and resources, contains information on citations and licenses, and offers some limited tools for querying the data. We then describe an R package designed to expedite work with IRW data.

#### The IRW website

The IRW website^8^ is built on a stack designed to facilitate data-sharing (Braginsky et al., In prep) and used previously for other data-sharing efforts (Frank et al., 2017; Sanchez et al., 2019; Zettersten et al., 2023). We describe the key features of the stack below.

- The IRW website is written in Quarto (https://quarto.org/). This platform allows for built-in Jupyter notebooks, which allows for us to build capacity for analysis directly into the website.
- We use Observable (https://observablehq.com/) for data visualizations. Observable allows us to produce custom visualizations with interactive elements to enable users to explore elements of the IRW directly on the website.
- The website is hosted via GitHub Pages (https://pages.github.com/). This platform allows for automatic rebuilding of the website upon changes to the underlying codebase.
- The data backend for the IRW website is Redivis (https://redivis.com/). Given that Redivis offers a suite of API tools, users can both download individual tables as needed and also take advantage of programmatic access to the full data resources of the IRW in common statistical computing environments such as R or Python. We discuss the API in more detail below. Redivis also manages data versioning, an essential feature for purposes of replicability.

This stack allows us to share the data in a way that is meant to be user-friendly. For example, users can directly browse through key summaries of individual tables (as well as the response-level data) using the website and then download those of interest.

**Fig. 1:**
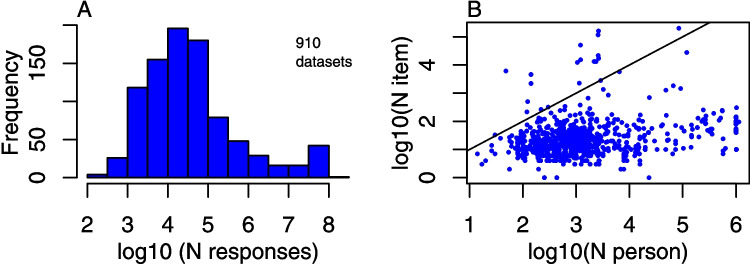
Overview of the size of the IRW. **A** A histogram showing the frequency of the $\log _{10}$ of the number of item responses in each table. **B** A scatter plot (in $\log _{10}$ scale) showing the number of persons and items in each table. The *black line* is 45 degree line

#### Programmatic work with IRW data in R

To support programmatic access to IRW tables, we provide an R package, irw. Key functions include:

- irw_filter(): This function can be used to identify tables with specific features (e.g., response time) or based on specific metadata values.
- irw_fetch(): Once a set of tables of interest has been identified, they can be retrieved by name using this function; tables will be returned as tibbles (Wickham et al., 2019).
- irw_long2resp(): Many use cases for IRW tables will require that the data be reformatted as a ‘wide’ matrix (with rows representing the object being measured and the columns the measurement probes). To facilitate this translation, we have created this function, which is meant to also provide some security so that, for example, responses that come from repeated waves of data collection are handled consistently.
- irw_save_bibtex(): We strongly encourage that researchers cite the tables that they use. To facilitate this, this function produces citations based on table names.

An example workflow illustrating these functions is shown below. Full documentation, including reference pages for all functions and usage examples, is available.^9^

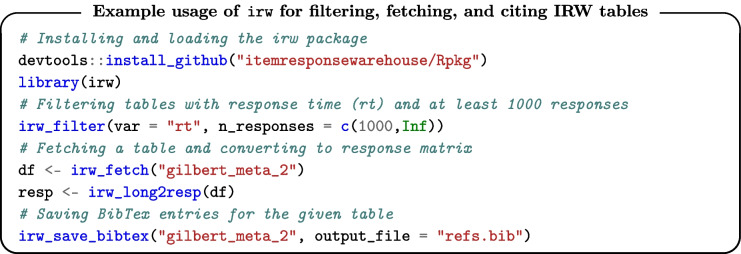



## A snapshot of the current data resources of the IRW

As of Version 28.2, the IRW contains 910 tables. A descriptive overview of the sizes of data represented in this version of the IRW is shown in Fig. 1 Panel A; the facts about the IRW that we describe here will evolve as more data is added to the IRW. This panel shows a histogram of the number of responses across all current IRW tables. Some tables are quite small, containing around $10^2=100$ responses. Most are much bigger: the me-dian table contains approximately $10^{4.4} \approx 24,000$ responses. Panel B shows a scatter plot of the number of persons (i.e., id identifiers) by the number of items (i.e., item identifiers). The majority of the tables involve fewer items than measurement objects, but there are a few tables containing very large numbers of items. Note that we typically limit tables to one million respondents (based on a random sample from the full set); if researchers are interested in the complete datasets, they can use the provided code that reformats the data to construct an IRW-compatible table with all respondents (which could then be analyzed alongside other IRW data resources).

A key point of variation among the IRW tables concerns density. Suppose Table 1 is translated into a conventional or ‘wide’ item response table where rows represent people and columns represent items. In the simplest case, every person responds to every item; this scenario is relatively common in our data. We index density by the proportions of cells in such a table that are not empty; in this simple case, density is 1 (as 100% of the cells have valid responses). In many cases, individuals do not respond to all items, and density may be less than 1. Common reasons for such density might be due to respondent behavior – e.g., respondents skipping items – or due to assessment design – e.g., balanced incomplete booklets (wherein respondents are only meant to take a small number of items) or adaptive assessments (in which case external information on items is typically required to obtain accurate ability estimates). Of the tables in the IRW, 27% have density values less than 1, but only 10% of these tables have density values less than .5. In other cases, respondents may attempt items multiple times, leading to density greater than 1. This commonly occurs in psychological testing when respondents make repeated attempts at a trial (especially when response time is the critical outcome rather than accuracy alone) and intensive longitudinal or experience sampling designs (Hamaker and Wichers, 2017; Asparouhov and Muthén, 2020; McNeish et al., 2021; Stieger and Kuhlmann, 2018). Of the current IRW tables, 24% have density values greater than 1.

**Fig. 2:**
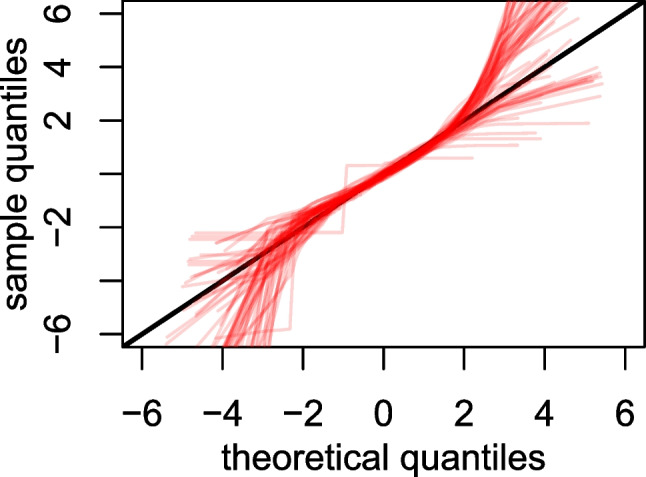
Lognormality of response time data from $N=68$ tables in the IRW that contain response time

## Brief tutorial for accessing and analyzing the IRW in R

The web interface can be used to get some understanding of the data in the IRW and to download individual files of interest. Larger-scale work, however, may benefit from alternative means of accessing the IRW data. In particular, heavy users of the IRW will benefit from the use of the custom R package irw for working with IRW data. In particular, we are incorporating a variety of techniques for filtering and fetching data from the IRW in this package.

### Standard psychometric analysis

Below, we demonstrate how straightforward analysis can be performed on a sample table (taken from Kim et al. , 2023). We conduct analysis using functions from a variety of commonly used R packages (Chalmers, 2012; Dinno, 2018; Revelle, 2024) to compute reliability, conduct dimensionality analysis, and then estimate factor analysis and IRT models.

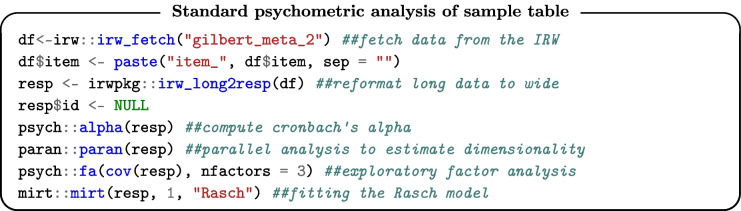



### Response time analysis

We now turn to a more complex example.^10^ In this example, we first identify all data with response time (i.e., a rt column), download the data, and analyze response times. This illustration could serve as a template for how to identify specific types of data in the IRW. We analyze 68 tables with response time in the IRW.^11^

We conduct a straightforward descriptive analysis of this data: we consider the degree to which the response time data for all persons and items appear lognormal. The lognormal model (e.g., a response of 10s is transformed to $\log (10)=2.3$) is a popular model for response time (van der Linden, 2006). Rather than formal tests (Sinharay and van Rijn, 2020) assessing whether the response times are lognormal, we focus on descriptive analyses (after restricting analysis to responses less than 30 minutes) using QQ plots, see Fig. 2. In general, the lognormal assumption seems to match the data fairly well through roughly the $z \in \{-2,2\}$ range. Outside of that range, there is clearly variation in the kurtosis of many distributions that would be inconsistent with the lognormal assumption.

### Analysis of item-level treatment effects

**Fig. 3:**
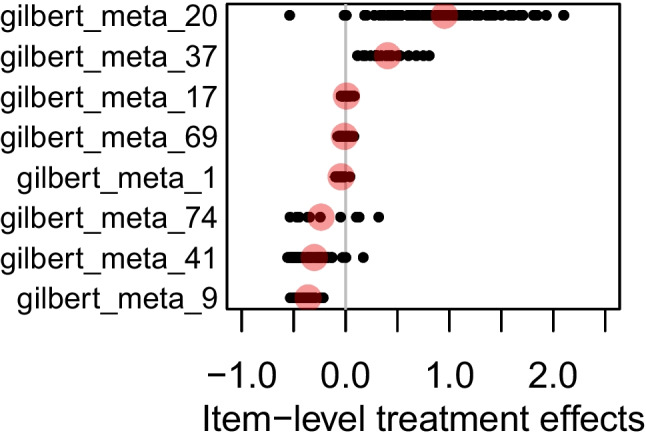
Item-level heterogeneous treatment effects on the logit scale: Overall treatment effect in *red* with item-level effects shown as *black* dots

There has been recent interest in analyzing the degree to which there is variation in item-level treatment effects of randomized experiments (Ahmed et al., 2023; Gilbert et al., 2023b, 2024c, d; Halpin and Gilbert, 2024; Gilbert et al., 2024e; Sales et al., 2021). Data in the IRW from such experiments have a treat column that indicates whether a respondent with a given id was a member of a treatment group. We offer a brief example of such analysis using 8 RCT tables (Banerjee et al. , 2017; Carpena , 2024; Cilliers , 2023; Duflo et al. , 2024; Gersten et al. , 2019; Gilbert , 2023a; Gilbert et al. , 2024a; Hidrobo et al. , 2016).^12^ As in earlier work (Ahmed et al., 2023; Gilbert et al., 2024c), we demonstrate pronounced variation across RCT outcomes in item-level heterogeneity with respect to treatment sensitivity (Fig. 3). In one table (gilbert_meta_69, Banerjee et al. , 2017), we see quite limited variation. In this case, the effect caused by the treatment is fairly consistent across the outcome’s items. In other cases (e.g., gilbert_meta_20, Gersten et al. , 2019), there is clear variation around a general effect of treatment. At present, the IRW currently contains over 95 tables from RCTs, which may be useful for research synthesizing causal inference and psychometric methods.

## Discussion

With the IRW, we aim to foster the development of a community resource that contains standardized and accessible item response datasets. In this paper, we have described the data available in the IRW, how it is standardized, and given examples of how to use and analyze this data. While development is ongoing, there is already a large volume of tables available to researchers in the IRW.^13^ These data are standardized, have information related to reuse and citation readily available, and can be pulled into the computing environments used by many psychometrics researchers.

There are limitations of the IRW data. The datasets used here should be viewed as potentially compromised due to choices made for the purposes of standardization. Decisions made for standardization and coherent analysis, given the generic interests of IRW researchers, may depart from the original intent of the data source (e.g., in general, we made decisions about how to define ‘items’ when there was some ambiguity that resulted in relatively few items rather than relatively more). While data in the IRW are easily accessible, they may not be authoritative, and researchers should consult the primary sources for each data source to verify the interpretation of the data (e.g., the work in Gilbert et al. (2025) provides one example of how this could be done, as the authors supplement data drawn from the IRW with replication materials from the original sources to explore item wording effects). We view the accessible standardized versions of these datasets in the IRW as an important resource despite this limitation.

Our ultimate goal is to make the IRW easy to use for researc-hers in psychometrics, psychology, educational measurement, and behavioral research. We aim to make it convenient to readily consider the performance of a hypothetical psychometric model in a wide array of data. Being able to readily probe variation in performance across a variety of datasets will make the generalizability of a given model’s use cases much more immediately apparent. Being able to consider such questions will, we hope, both push research in more productive directions and also lead to novel research questions.

## Data Availability

See https://itemresponsewarehouse.org.

## Footnotes

1 A note about nomenclature: we use ‘dataset’ to refer generically to data and ‘table’ to refer to the specific reformatted data resources available via the IRW.

2 https://github.com/itemresponsewarehouse/misc/tree/main/data

3 For clarity, note that we have begun to construct a separate repository of synthetic/simulated item response data resources, but they are not part of the original IRW and we do not discuss them here.

4 https://itemresponsewarehouse.org/standard.html

5 For this latter case, we have developed resources to help them do so, see irw::irw_merge() in the R package we describe in Section “Programmatic work with IRW data in R”.

6 For comparison, a ‘wide’ representation of this simple toy data might be $$ \begin{pmatrix} 0 & 0\\ 1 & 1 \end{pmatrix}$$ where the columns are items and the rows represent the subject of measurement.

7 https://www.unixtimestamp.com/

8 https://itemresponsewarehouse.org/

9 https://itemresponsewarehouse.github.io/Rpkg/

10 Code available at https://github.com/itemresponsewarehouse/misc/blob/main/manuscript_src/02_responsetime.R.

11 Data come from: Jones et al. (2024); Artistic (2023); Jorgenson (2016); Yarkoni (2010); Van Der Maas and Wagenmakers (2005); Cizek and Wollack (2016); Borst et al. (2011); Depression (2021); Suggate (2024); Settles (2018a, 2018b, 2018c, 2018d, 2018e, 2018f, 2018g, 2018h, 2018i); Exposure (n.d.); Belfi and Kacirek (2020); Goldberg (1992a, 1992b, 1992c, 1992d, 1992e); Brown et al. (2013); Hampshire et al. (2012a, 2012b, 2012c); Brotherton et al. (2013); Papoušek et al. (2016); Glatz (2023a, 2023b); Jastrzębski et al. (2022); Zhang et al. (2023); Development (n.d.); Inoue et al. (2024); Christie and Geis (1970); Ratcliff and Hendrickson (2021a, 2021b); Multifactor (n.d.); O’Brien and Yeatman (2020); Ratcliff and Hendrickson (2021c, 2021d, 2021e, 2021f); Much et al. (2025); Nisbet and Zelenski (2013); Hooper et al. (2024); Archambeau et al. (2022); Richmond et al. (2003); Bohn et al. (2023); Pisa (2017a, 2017b, 2017c, 2019a, 2018cb, 2019c, 2019d, 2023a, 2023b, 2023c); Mirels and Garrett (1971); Yeatman et al. (2021); Ratcliff and Rouder (1998); Bendall et al. (2024); Duñabeitia (2021); Bohn et al. (2022).

12 Code available at https://github.com/itemresponsewarehouse/misc/blob/main/manuscript_src/03_il-hte.R.

13 Note that we welcome contributions to the IRW and describe how users can contribute data to the IRW at: https://itemresponsewarehouse.org/contribute.html.

## Code Availability

Relevant materials are available at https://github.com/itemresponsewarehouse.

## Declarations

### Open practices statement

The data and materials for all experiments are available.
